# Proteomic Analysis of Barley (*Hordeum vulgare* L.) Leaves in Response to Date Palm Waste Compost Application

**DOI:** 10.3390/plants11233287

**Published:** 2022-11-29

**Authors:** Emna Ghouili, Ghassen Abid, Moez Jebara, Rim Nefissi Ouertani, Ana Caroline de Oliveira, Mohamed El Ayed, Yordan Muhovski

**Affiliations:** 1 Laboratory of Legumes and Sustainable Agrosystems, Centre of Biotechnology of Borj-Cedria, (L_2_AD, CBBC), P. B. 901, Hammam-Lif 2050, Tunisia; 2 Laboratory of Plant Molecular Physiology, Centre of Biotechnology of Borj Cedria, BP 901, Hammam-Lif 2050, Tunisia; 3 Biological Engineering Unit, Department of Life Sciences, Walloon Agricultural Research Centre, Chaussée de Charleroi, BP 234, 5030 Gembloux, Belgium; 4 Laboratory of Bioactive Substances, Centre of Biotechnology of Borj Cedria, BP 901, Hammam-Lif 2050, Tunisia

**Keywords:** barley, compost, LC-MS/MS, leaves, proteomics, qRT-PCR

## Abstract

Composts are an emerging biofertilizers used in agronomy that can improve crop performance, but much less is known regarding their modes of action. The current study aimed to investigate the differentially abundant proteins (DAPs) in barley leaves associated with growth promotion induced by application of date palm waste compost. Morphophysiological measurements revealed that compost induced a significant increase in plant height, chlorophyll content, gas exchange parameters and plant biomass. LC-MS/MS analyses indicate that compost induced global changes in the proteome of barley leaves. A total of 62 DAPs (26 upregulated and 36 downregulated) among a total of 2233 proteins were identified in response to compost application. The expression of DAPs was further validated based on qRT-PCR. Compost application showed altered abundance of several proteins related to abiotic stress, plant defense, redox homeostasis, transport, tricarboxylic acid cycle, carbohydrate, amino acid, energy and protein metabolism. Furthermore, proteins related to metabolic processes of phytohormone, DNA methylation and secondary metabolites were induced. These results indicate that barley responds to compost application by complex metabolism pathways and may result in a positive alteration in a physiological and metabolic barley plant state which consequently could lead to improved growth and stress adaptation observed in compost-treated plants.

## 1. Introduction

Fertilizer application plays a pivotal role in increasing agricultural production. With the increasing demand for sustainable and environmentally friendly agricultural production, including organic farming, the orientation towards integrating biostimulants and organic fertilizers such as compost, biochar and vermicompost into agriculture management has become more crucial. Organic fertilizers are an important source of nutrients for agricultural production since their application helps maintained soil nutrient balance and promoted their absorption, improved soil structure, soil physical and chemical properties, increased soil organic matter which leaded in increasing growth and quality of crops.

In general, composts are mainly produced from agricultural byproducts derived from animal and plant sources. They contain many plant essential nutriments (macro- and microelements), as well as numerous active minerals and organic compounds that includes phytohormones, amino acids, polyamines, carbohydrates and other signalling molecules such as humic substances [1]. Compost is known to have a wide range of beneficial effects on plant growth promotion and development and soil fertility due to their high nutrient and organic matter content [2]. Furthermore, compost enhanced the antioxidant content in plants and consequently improved plant defense against pests and diseases [3] and alleviated the negative effects of environmental constraints such as drought and salinity which lead to promote crop growth, development, yield, and quality [4].

Although compost’s direct benefits on plant productivity and quality have been clearly documented and extensively investigated and various morphological, agronomical and physiological characterizations have been implemented, the molecular mechanisms underlying this effect remain unclear.

The compost effects are complicated due to the heterogeneous nature of the raw materials used for compost production and to a set of actions performed by enormously active substances that are difficult to describe separately [5]. Therefore, understanding the molecular mechanisms related to the mode of action of compost is indispensable to improve the development of better practices of compost applications as a plant bio-fertilizer.

Interestingly, recent scientific progress in plant-omics, including genomics, transcriptomics, proteomics, and metabolomics, provides us with essential high-throughput tools capable of identifying global changes in plant response to external stimuli such as abiotic and/or biotic stresses [6]. Several studies reported that the application of biofertilizers and/or biostimulants affected the expression of several genes serving as key regulators of different metabolic pathways; some of them involved in photosynthesis, detoxification, transport, oxidative stress response and energy production mechanisms [7]. In this context, the use of proteomics analyses which contribute to the actual functionality of regulatory reactions and cascades will provide more direct information about the biofertilizers’ mechanism of action than functional genes and even the corresponding messenger RNAs.

Protein profiling of Arabidopsis roots treated with humic substances (HS) revealed stimulation of proteins involved in energy metabolism which is required for plant growth. Moreover, regulation of enzymes involved in redox homeostasis suggests a pivotal role of reactive oxygen species (ROS) in response to HS stimulus, possibly acting as signalling molecules to coordinate metabolic processes like plant growth and development [8]. Thus far, much less information is available about the mechanism underlying the effect of compost as a biofertilizer on plant growth and its promotion up to molecular level.

Recently, application of date palm waste compost has positively affected plant growth, biomass accumulation, plant development, yield and yield components of barley crops under a field organic farming system [9]. Moreover, barley plant growth promotion due to compost application as biofertilizer was associated with the expression of different genes involved in nutrient uptake and transport in both root and leaf tissues, indicating their potential role in increasing nutrient uptake, assimilation and translocation capacity of barley plant under organic fertilizers [9]. It is therefore important to gain a better understanding regarding the positive effect of date palm compost on the growth of barley using omics approaches for delineating molecular mechanisms of biological processes. Mass spectrometry (MS)–based high-throughput proteomics is the core technique for large-scale protein characterization which could explain the variation in proteome profile due to date palm waste compost application through the comparative in silico analysis with other proteins curated from plant protein sequence databases [10]. Thus, the presence of specific proteins in plant samples is a reliable indicator of the effect of compost applied.

Leaves constitute the basic organ of photosynthesis and are eminently important vegetative organs supplying the energy, nutrition and hormones for the plant growth, biomass accumulation and development. Therefore, in the current study LC-MS/MS analysis was performed to understand the molecular mechanisms underlying the effects of date palm waste compost on total protein expression in the leaves of barley plants. Moreover, this work will provides comprehensive information which contributes to a better usage of date palm waste compost as a plant biofertilizer used in crop production.

## 2. Results

### 2.1. Growth Traits and Physiological Parameters

At the tillering stage, vegetative activity which is evaluated as shoot length (SL) and shoots dry weight (SDW) was higher in barley plants subjected to compost fertilisation compared to non-treated plants (Supplemental Figure S1). The SL and SDW increment was equal to 29% and 63%, respectively in barley plants amended by compost (Table 1). Moreover, barley plants which received compost showed higher photosynthetic pigment concentrations when compared to non-amended barley plants. The chlorophyll a (Chla), chlorophyll b (Chlb), total chlorophyll (Chlt) and carotenoids increases were 21%, 15%, 17% and 118%, respectively in comparison to the control. In this context, compost treatment also had a significant effect on gas exchange parameters. The addition of 30 t ha^−1^ of date palm waste compost increased the net photosynthetic rate (Pn), stomatal conductance (gs), transpiration rate (E) and internal concentration of CO_2_ (Ci) by 90%, 77%, 67% and 6%, respectively in comparison to the control plants (Table 1).

### 2.2. Identification of Differentially Abundant Proteins (DAPs) in Response to Date Palm Waste Compost Application

To understand the proteomic response of barley to compost application, changes in the leaves of the barley plants under date palm waste compost (30 tha^−1^) treatment were detected. In this study, we used LC-MS/MS to perform quantitative analysis of protein by using an Eksigent nanoLC Ultra 2D nanoHPLC (SCIEX) coupled to a Q Exactive PLUS mass spectrometer (Thermo, Waltham, MA, USA). A total of 2233 proteins were identified in control and treated samples with a FDR of 0.17% for peptides and 0.08% for proteins (Supplemental Table S1). The proteins identified covered a wide range of biological processes. Using a stringent threshold of fold changes (cutoff of over 1.5 for increased expression and less than 1/1.5 (0.67) for decreased expression) and *p*-value < 0.01, a total of 62 proteins were determined to be differentially expressed in compost-treated samples compared to control samples, of which the abundance of 26 proteins increased while the abundance of the other 36 proteins decreased (Table 2).

### 2.3. Biological Process, Molecular Function, Protein Class and Subcellular Localization of DAPs

**Figure 1:**
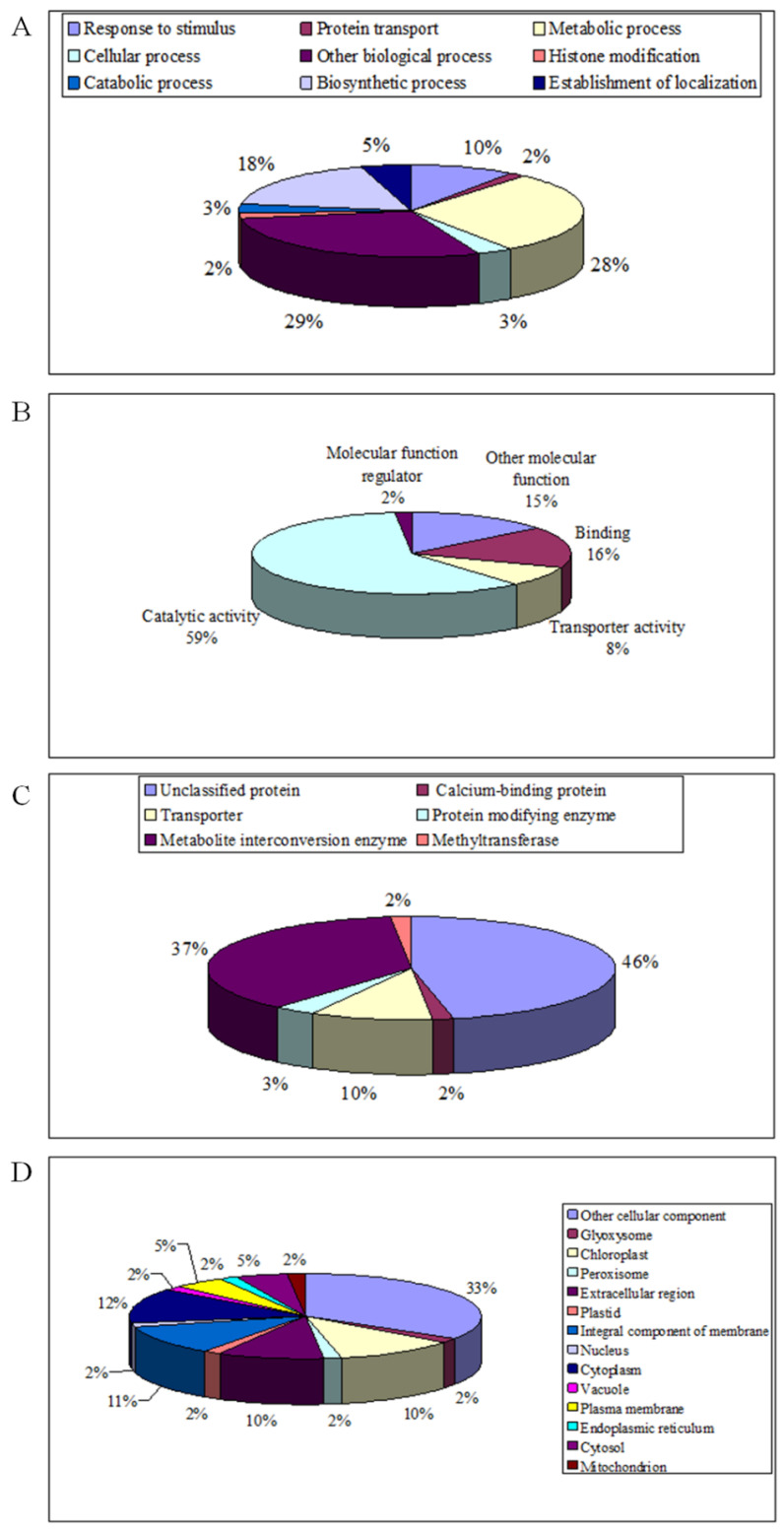
Categories of differentially abundant proteins (DAPs): biological function (**A**) molecular function (**B**), protein class (**C**) and subcellular localization (**D**).

The 62 DAPs were grouped into 9 functional categories based on the biological processes according to the published literature and UniProt online analysis (https://www.uniprot.org/, accessed on 5 March 2022) (Figure 1A). They are involved in response to stimulus (10%), metabolic process (28%), biosynthetic process (18%), protein transport (2%), cellular process (3%), histone modification (2%), catabolic process (3%), establishment of localization (5%) and other biological process (29%). In addition, all DAPs identified in leaves were classified by molecular function into 5 categories (Figure 1B) such as catalytic activity (59%), transporter activity (8%), binding (16%), molecular function regulator (2%) and other functions (15%). Based on protein class, these 62 DAPs were classified into 6 categories (Figure 1C), including transporter (10%), protein modifying enzyme (3%), metabolite interconversion enzyme (37%), methyltransferase (2%), calcium-binding protein (2%) and unclassified protein (46%). Thus, these results indicate that the DAPs in response to date palm waste compost application were involved in diverse biological processes and multifarious functions.

The subcellular localization analysis (Figure 1D) showed that these proteins were mainly localized in the chloroplast (10%), cytoplasm (12%), cytosol (5%), extracellular region (10%), plastid (2%), integral component of membrane (11%), plasma membrane (5%), glyoxysome (2%), vacuole (2%), nucleus (2%), endoplasmic reticulum (2%), peroxisome (2%), mitochondrion (2%) and other cellular components (33%).

### 2.4. KEGG Pathway Enrichment Analyses of DAPs

Kyoto Encyclopedia of Genes and Genomes (KEGG) pathway analysis was also performed in order to identify pathways regulated by date palm waste compost application based on enrichment analysis of the DAPs (Table 2). A total of 17 proteins related to metabolic pathways were identified. Among them, 6 were upregulated and 11 were downregulated. Among the 11 proteins involved in biosynthesis of secondary metabolites under compost treatment, 5 were upregulated and 6 were downregulated. Furthermore, 5 proteins were involved in amino acid-related metabolism, with 4 of them upregulated and one downregulated. More than 30% of proteins were identified as differentially expressed proteins corresponding to other pathways. Among them 6 were upregulated and 15 were downregulated. In addition, 4 proteins related to citrate cycle (TCA cycle), MAPK signalling pathway, carbon metabolism and protein processing were identified and all of them were upregulated under compost treatment. There were 2 proteins related to ABC transporters and ubiquitin mediated proteolysis and they were all downregulated under compost treatment. Two proteins related to energy metabolism were identified and one of them was upregulated, while the second was downregulated. Thus, these results indicate that the upregulated proteins were significantly enriched in carbon metabolism, biosynthesis of amino acids, protein processing, MAPK signalling pathway, biosynthesis of secondary metabolites, metabolic pathways, energy metabolism and citrate cycle (TCA cycle). The downregulated proteins were significantly enriched in ubiquitin mediated proteolysis, ABC transporters, biosynthesis of secondary metabolites, metabolic pathways and energy metabolism.

### 2.5. Protein Profile Validation by Quantitative Real-Time Polymerase Chain Reaction (qRT-PCR)

In order to validate the changes of protein levels identified in the proteomic analysis, a quantitative real-time PCR analysis of 14 genes randomly picked was performed (Table 3).

**Figure 2:**
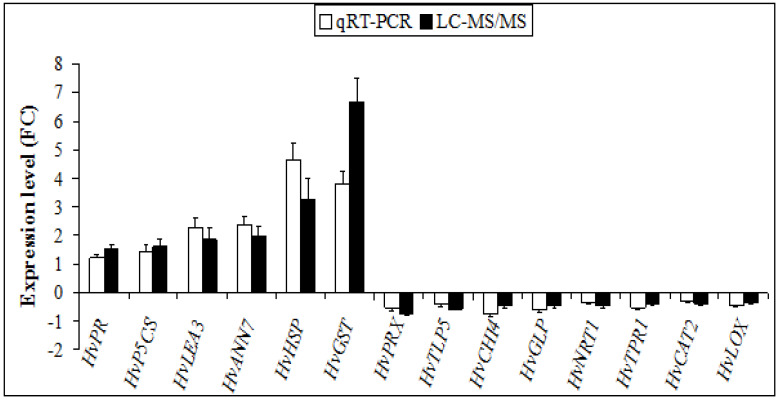
Changes in transcript levels and their protein abundance in response to compost treatment in barley leaves.

Results showed that the mRNA levels of *HvPR*, *HvP5CS*, *HvLEA3*, *HvANN7*, *HvHSP* and *HvGST* showed upregulation to compost treatment, while *HvPRX*, *HvTLP5*, *HvCHI4*, *HvGLP*, *HvNRT1*, *HvTRP1*, *HvCAT2* and *HvLOX* downregulated (Figure 2). In general, the variation trends of these genes under compost treatment were in good accordance with the proteomic analysis results. Indeed, two genes *HvHSP* and *HvGST* showed little correlation between their transcript and protein quantities which could be due to the variation in protein post-translational modification, while 12 genes showed a similar trend between the two. Moreover, for supporting the reliability of LC-MS/MS analysis data, a correlation coefficient (R^2^) (of the fold changes between qRT-PCR and LC-MS/MS values) of 81% was obtained (Supplemental Figure S2), indicating that the results of qRT-PCR were consistent with those derived from the proteomics analysis.

### 2.6. Protein–Protein Interaction (PPI) Network Analysis of DAPs

**Figure 3:**
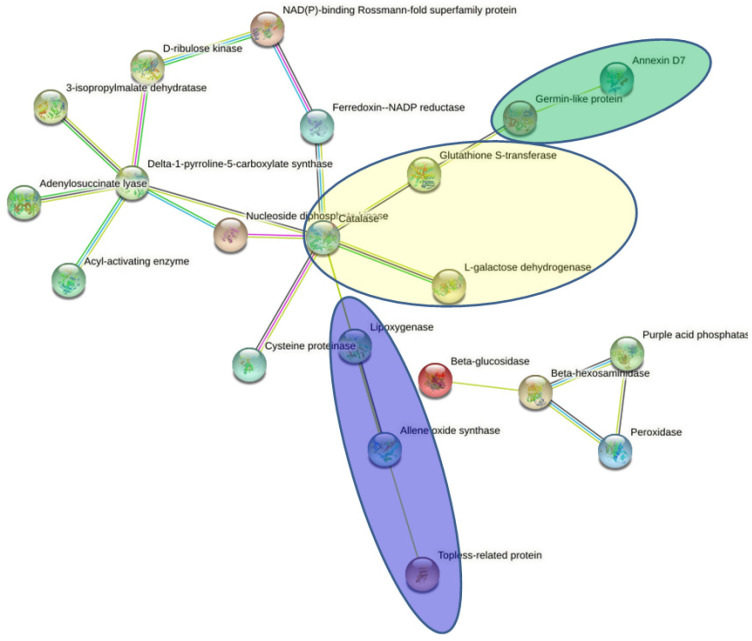
Protein–protein interaction (PPI) network analysis among the significantly expressed proteins in barley leaves under date palm waste compost treatment using STRING database. DAPs related to redox homeostasis, plant defense and phytohormone action were indicated in backgrounds of yellow, green and blue, respectively.

A PPI network was generated using the STRING database to highlight the relationship of the 62 DAPs in response to compost treatment (Figure 3). Twenty-one proteins out of the 62 identified in this work comprise the interaction network. However, 41 proteins including protein markers involved in plant defense response such as pathogenesis-related protein (PR), 23 kda jasmonate-induced protein, Chitinase IV and thaumatin-like protein (TLP5) as well as proteins involved in environmental stress response including heat shock protein (HSP) and late embryogenesis abundant protein (LEA3) have no functional relationship to each other and with other proteins. Hub analysis of the network suggested peroxidase, catalase, delta-1-pyrroline-5-carboxylate synthase, ferredoxin-NADP reductase, L-galactose dehydrogenase, glutathione S-transferase, topless-related protein, lipoxygenase and allene oxide synthase were central proteins in these networks in the barley plant response to compost treatment. Furthermore, in the network of interactions, some proteins were found to play core roles in the barley defense system against diseases, which included annexin D7 and germin-like protein

In the protein interaction network, for antioxidant defense system, interaction was observed with catalase, which in turn interacted with L-galactose dehydrogenase, ferredoxin-NADP reductase, cysteine proteinase, nucleoside diphosphate kinase and glutathione S-transferase, which further interacted with annexin D7 and germin-like protein. These proteins are involved in antioxidant defense, detoxification, photosynthesis and protein homeostasis.

Delta-1-pyrroline-5-carboxylate synthase had interactions with 3-isopropylmalate dehydratase, adenylosuccinate lyase, acyl-activating enzyme and D-ribulose kinase, which further interacted with NAD(P)-binding Rossmann-fold superfamily protein. These proteins are involved in biosynthesis of amino acids, carbon metabolism, biosynthesis of secondary metabolites and metabolic pathways. Apparently, three protein–protein interaction networks which related to redox homeostasis, phytohormone action and plant defense networks were significantly enriched.

### 2.7. Proposed Model of a Possible Mode of Action of Compost in Barley Plants at the Tillering Stage

**Figure 4:**
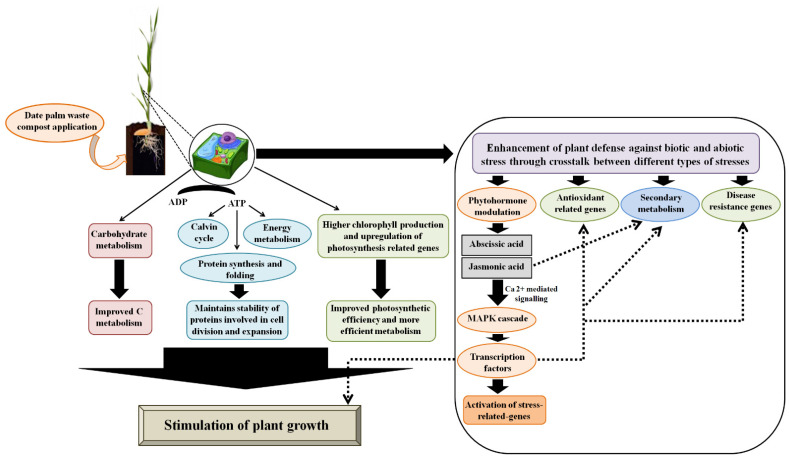
Model for mode of action of date palm waste compost in promoting growth of barley plants.

Based on the annotated biological functions and the relevant published literature on the key DAPs identified in the current study, a hypothetical model that represents a possible mode of action of compost in barley plants growth promotion at the tillering stage was developed (Figure 4). Date palm waste compost can promote plant growth via regulation of proteins related to photosynthesis, protein metabolism and energy metabolism. At the same time, compost by virtue of phytoactivators contained in its formulation (e.g., humic substances, phenols and amino acids) can modulate the level of stress-related hormones, thus activating a cascade of events that ultimately causes the regulation of both proteins involved in redox homeostasis, biosynthesis of secondary metabolites, defense against biotic and abiotic stress, growth and development.

## 3. Discussion

### 3.1. Application of Date Palm Waste Compost Promote Growth of Barley Plants

Several studies reveal that application of date palm waste compost could be used as an efficient organic fertilizer for growth, yield and quality of crop plants such as barley [9], alfalfa [11] and tomato [12]. Compared with untreated soil, the date palm waste compost application significantly improved vegetative activity of the barley plant which is evaluated as shoot length and shoot dry weight. This could be due to the compost richness in easily assimilated available substances, providing carbon, nitrogen, phosphorus, and potassium to soil [13]. The positive effect of compost amendment on barley growth has been also demonstrated by other studies [9,14] who reported that compost application increased plant height and dry biomass. Furthermore, barley plants which received 30 t ha^−1^ of date palm waste compost showed higher photosynthetic pigment (Chla, Chlb, Chlt and carotenoids) concentrations when compared to the non-amended barley plants. Indeed, the photosynthetic pigments increment registered in barley plants subjected to compost exhibited photosynthetic performance improvement following this organic application. In this perspective, several studies noticed an improvement in chlorophyll content and photosynthesis parameters in wheat leaves after compost application [4,15].

### 3.2. Application of Date Palm Waste Compost Altered Barley Leaf Protein Profiles

To the best of our knowledge, the effects of date palm waste compost on the proteome of barley plants have not been investigated yet. In the present study, to investigate the barley plant response to date palm waste compost application, a proteomics analysis was performed using an LC-MS/MS approach. Moreover, this study is also the first of its kind to integrate physiological results with proteomics data to understand the responses triggered by date palm waste compost in barley leaves at the molecular level.

The proteomic analysis suggests that compost treatment induced global changes in the proteome of barley leaves.

A total of 62 DAPs were identified under compost treatment in barley leaves. The DAPs were classified into different categories including signalling, photosynthesis, plant stress and defense response, osmotic regulation, protein synthesis and turnover, ROS scavenging, carbohydrate and energy metabolism, cell structure and cell cycle, lipid metabolism and amino acid metabolism (Table 2).

### 3.3. Proteins Involved in Redox Homeostasis

In this study, some DAPs that are unequivocally related to redox homeostasis were identified in barley leaves in response to compost treatment such as L-galactose dehydrogenase (LGALDH), glutathione S-transferase (GST) and catalase (CAT). The abundance of GST and LGALDH increased significantly under compost treatment. This implies that there could be a direct or indirect triggering of ROS in barley leaves treated with compost, as also reported previously in forage river saltbush (*Atriplex amnicola* Paul G. Wilson) [16]. This meant that this triggering of the antioxidative system is one of the vital upstream events that could be regulating a cascade of other downstream processes [17]. Similarly, several studies reported the induction of genes involved in redox homeostasis and antioxidative system by the application of other classes of biostimulants such as protein hydrolysate (PH) in tomato [18], humic substances (HS) and seaweed extracts in Arabidopsis [19,20] and maize [21]. In this study, the abundance of CAT was significantly decreased under compost treatment. Similar results have also been recorded in the case of wheat plants treated with a free amino acid biostimulant [22]. The alteration in the abundance of antioxidant enzymes in response to HS treatment has also been reported in Arabidopsis [8]. These authors reported the increase abundance of disulfide-isomerase 2, while GST and superoxide dismutase (SOD) were down-regulated by HS treatment. In general, low ROS levels are necessary for regulation of several basic biological processes such as normal plant growth, cellular proliferation and differentiation, hormone signalling and improving plant tolerance to biotic and abiotic stress [23]. Thus, the induction of an antioxidant system may fortify plants against different future abiotic and biotic stresses [24]. In the present study, the compost amendment has led to a significant improvement of the antioxidant defense mechanism, and this may be one of the conserved mechanisms by which compost acts on barley plants. The positive effect of other classes of biostimulants including HS in redox capacity has been also demonstrated in several plant species like maize [17] and tomato [25]. Taken together, in response to compost treatment several DAPs related to redox homeostasis were identified. This implies that there could be a direct or indirect triggering of ROS in barley leaves treated with compost. Hence, we suggest this triggering of ROS-processing or antioxidative system to be one of the vital upstream events that could be regulating a cascade of other downstream processes.

### 3.4. Proteins Involved in Stress Response

In the current study several DAPs related to stress defense were identified such as pathogenesis-related protein (PR1), late embryogenesis abundant protein (LEA3), Annexin D7, 23 kda jasmonate-induced protein, heat shock protein (HSP), thaumatin-like protein (TLP5), chitinase IV (CHI4), germin-like protein (GLP), peroxidase (PRX), lipoxygenase (LOX), topless-related protein 1 (TPR1) were differentially regulated which is consistent with different biostimulants previously reported including HS, PH, seaweed extracts and microorganism-based biostimulants [26]. Biostimulants stimulate the expression of stress-related genes resulting in regulating and modifying morphological and physiological processes in plant which eventually lead to abiotic and biotic stress tolerance or resistance, promote plant growth and development, and improve quality and yield of crops [27,28]. In the present study, accumulation of protective proteins such as HSP and LEA3 is expected to be beneficial for mitigating environmental stresses. Higher concentrations of these proteins were also identified in durum wheat treated with two foliar biostimulants, seaweed extract DPI4913 and fungal extract AF086 [29]. HSP function as molecular chaperones that play an important role in stabilising proteins under both optimal and stress conditions. They facilitate protein folding, degradation, complex assembly, translocation, reducing the intracellular level of reactive ROS and maintaining membrane integrity [30]. Expression patterns of late embryogenesis abundant (LEA) gene revealed that is a multi-functional stress protein to maintain normal metabolism for higher plants under stress conditions, and its gene can code RNA-regulatory-proteins, which can regulate related events, such as gene expression and development [31]. Furthermore, compost increased the abundance of Annexin D7 (ANN7) which plays a potential protective role against oxidative stress and maintains cell redox homeostasis. It is well known that plant annexins are a family of calcium-dependent phospholipid-binding proteins that regulate diverse aspects of plant growth, development, and stress responses [32,33]. Moreover, overexpression of *Brassica juncea* annexin gene in the tobacco plant enhanced resistance not only to mannitol, NaCl, CdCl_2_ and H_2_O_2_ but also to the oomycete pathogen (*Phytophthora parasitica* var. nicotianae) in transgenic lines, suggesting that annexin could provide tolerance to a variety of abiotic and biotic stresses [34].

These results are in agreement with the observed effect of biostimulants such as humic acid (HA) on leaf protein expression [35]. Indeed, date palm waste compost appeared to increase the expression of some proteins involved in abiotic stress tolerance.

Promoting and eliciting plant defenses is another possible role of many biostimulants including plant growth promoting rhizobacteria (PGPR) and HS through regulation of several genes sensitive to important phytohormones involved in modulating herbivore-induced plant defenses like jasmonic acid, ethylene and salicylic acid [36].

Pathogenesis-related proteins (PRs) are specific plant proteins induced by biotic stress and they play an important role in plant defense against various types of pathogens. According to their sequence homology, molecular structure and enzyme activity, PR proteins have been classified into 17 different families including PR1 (antioomycete and antifungal), PR2 (β-1,3-glucanases), PR3 (chitinases), PR4 (antifungal), PR6 (proteinase inhibitors), defensins, glycine-rich proteins, osmotinlike proteins, proteinases, thaumatine-like proteins (TLP), cysteine-rich proteins, lysozymes, chitosanases, lipoxygenases, peroxidases and thionins [37]. Previous research reported differential expression of plant defense proteins in response to the application of biostimulants. Foliar application of chitosan induced chitinase, PR2 and lipoxygenase (LOX) in potato and tomato infected by *Phytophthora infestans* or nematodes [38]. In this context, the application of *Ascophyllum* extract increased the expression of PR1, PR5, chitinase, lipid transfer protein (LTP), phenylalanine ammonia lyase (PAL), chalcone synthase and non-expressing pathogenesis-related protein (NPR1), resulting in reduction of Alternaria and Botrytis foliar blight disease incidence levels in greenhouse-grown carrots [39]. Application of biostimulant prepared from combination of brown macroalga *Ascophyllum nodosum* (Liquid Seaweed Extract; LSE) and chitosan increased the expression of PR1, PR2 and chitinase which lead to reduced *Fusarium* head blight (FHB) and mycotoxin contamination in wheat [40]. In the current study, many proteins associated with plant defense responses and tolerances were differentially expressed in leaf tissue of barley under compost treatment. Indeed, PR1 protein and 23 kda jasmonate-induced protein were found to be increased, while thaumatin-like protein (TLP5), chitinase, lipoxygenase (LOX) and peroxidase (POX) were decreased. It is possible that the compost used in the study is the source of various elicitors’ compounds that promote the accumulation of salicylic acid (SA) and jasmonic acid (JA) and increase the expression of protein related to defenses against pathogens such as PR1 which is a reporter of the SA-dependent pathway and 23 kda jasmonate-induced protein (JIP-23) related to JA-dependent signalling pathway. The elevated expression of these proteins in the compost treated plants may suggest that SA and JA-dependant pathways in the challenged barley plants interfere with some defense mechanisms. Interestingly, in barley plants JA may regulate the development of seed through accumulation of JIP-23 which is a valuable indicator of endogenous JA levels and involved in sugar import into filling grain [41]. Moreover, the accumulation of PR1 in tobacco leaves has been related to defense against pathogens and specific physiological processes such as leaf senescence and floral development [42].

Plant germin-like proteins (GPLs) play key roles in plant growth and development along with other many physiological processes such as plant height, chlorophyll biosynthesis, fibre development, seed dormancy, dwarfism, high density and diversity of trichomes [43,44,45]. In cucumber, differential expression of some *CsGLP* genes during different developmental stages of ovaries, suggesting their possible regulatory roles in the ovary development of cucumber [46]. In addition, GLPs are also related to the responses of plants to biotic [46] and abiotic stress [47]. In a similar way, date palm waste compost showed increased abundance of GLP protein in leaf tissue suggesting a possibly important role in barley plant growth, development and stress response. Thus, the upregulation of several proteins related to stress response observed in compost-trated plants serves a confirmation that compost application enhanced stress tolerance via osmoprotection avenues.

### 3.5. Proteins Involved in Energy Metabolism, Photosynthesis and Chlorophyll Metabolism

The application of alfalfa hydrolyzate plants (AH) and red grape skin extract (RG) induced the expression and the activity of several enzymes involved in the tricarboxylic acid cycle (TCA cycle) in hot pepper plants [48]. Moreover, in maize roots, PH treatment induced changes in the abundance of proteins involved in the TCA cycle [17]. Consistent with the results in hot pepper and maize, compost treatment increased the abundance of Aconitase_C domain-containing protein involved in the TCA cycle in barley leaves. Furthermore, it was evidenced in our results that compost treatment had an effect on the other proteins related to carbohydrate (D-ribulose kinase) metabolism consistent with previous reports on PH [17]. The increased abundance of proteins related to glycolysis/TCA cycle and carbon metabolism suggests an enhanced energy demand and mobilisation of metabolites in order to maintain physiological activity in leaf tissues.

As the key process of primary metabolism, photosynthesis provides the energy needed for the plant growth and development. Regarding the pathway of the photosynthesis, the protein encoding photosynthetic NDH subunit of lumenal location 2, chloroplastic was upregulated. These findings suggest that compost treatment may lead to increased photosynthetic ability in barley leaves. Proteomic analysis revealed that magnesium chelatase (Mg chelatase) and magnesium-protoporphyrin IX monomethyl ester (oxidative) cyclase related to chlorophyll biosynthesis showed increased abundances under compost treatment. However, not all the protein changes under compost treatment are consistent with previous studies. Consequently, this may positively affect the enzymes involved in photosynthesis and the function of chloroplast under compost treatment. Thus, the upregulation of both proteins mentioned above might be the key elements contributing to enhancing photosynthetic pigment concentrations and photosynthetic activity, which can promote the growth of compost-treated plants. Overall, the energy produced from the TCA cycle and photosynthesis can fuel a wide-range of energy-demanding biochemical processes that aid in plant growth and development such as gene expression and metabolism.

### 3.6. Proteins Involved in Amino Acid Metabolism

Several proteins identified in this study are related to amino acids biosynthesis pathway such as delta-1-pyrroline-5-carboxylate synthase (5PCS), bifunctional enzyme aspartokinase/homoserine dehydrogenase (AK-HSDH), phospho-2-dehydro-3-deoxyheptonate aldolase, as well as the 3-isopropylmalate dehydratase, which are positively affected by compost treatment and their expression increased in barley leaf tissue. The increased abundance of proteins associated with biosynthesis of amino acids pathway was found in maize roots treated with PH [17]. 5PCS involved in proline biosynthesis that catalyzes glutamate to pyrroline-5 carboxylate, an intermediate in proline biosynthesis. Due to its osmoprotective properties, proline not only accumulates during abiotic stress, but also in different tissues of plants under non-stress conditions. Indeed, proline modulates a wide array of functions including cell wall elongation and modifications, xylogenesis, stem elongation, root, and shoot growth, inflorescence architecture, embryo formation/seed development, and seed germination, indicating a potential role in plant growth, differentiation and development [49]. AK-HSDH catalyses the first and the third steps toward the synthesis of the essential amino acids threonine, isoleucine, lysine and methionine. A previous metabolic study reported the accumulation of various amino acids including threonine, isoleucine, lysine and methionine in Arabidopsis [50] and perennial grass [51] under heat stress. Lysine was synthesised in the metabolic route of aspartate, which produces threonine, methionine, and isoleucine and involved through the sacharopine pathway (SACPATH) in response to biotic and abiotic stresses [52]. Methionine regulates the development of plants and assimilation of polyamines, secondary metabolites and ethylene which lead to improving tolerance against diverse environmental stresses through the S-adenosyl methionine (SAM) pathway [53]. Phospho-2-dehydro-3-deoxyheptonate aldolase is involved in the synthesis of aromatic amino acid derived from intermediates of the pre-chorismate pathway up to shikimate as well as, this enzyme being involved in the first critical step for the shikimate pathway and phenylpropanoid biosynthesis [54]. Accumulation of phenylpropanoids was revealed in PH-treated roots maize [17]. 3-isopropylmalate dehydratase performs the second step in the biosynthesis of leucine that plays an important role in controlling protein synthesis and regulating cell metabolism. Application of biostimulant based branched-chain amino acids (BCAA) leucine (L), isoleucine (IL), and valine (V) significantly increased creeping bentgrass plant growth parameters such as root weight and shoot density compared to control [55]. Therefore, the stimulation of barley plant growth (Table 1) shown here by date palm waste compost application could be explained by the supposed enhanced availability of amino acids used directly by the plant for growth [56]. Indeed, amino acids are crucial metabolites that interact with numerous branches of metabolism which stimulate plant growth [57].

Amino acids have various prominent functions in plants, mainly protein biosynthesis, necessary for plant growth and development. Among them, sulfur and nitrogen-containing amino acids act as key elements in primary protein structure. Besides their usage during protein biosynthesis, they also act as a precursor of a large number of multi-functional secondary metabolites and play pivotal roles during signaling processes [58]. Overall, compost induced the increase in abundance of some proteins involved in amino acid metabolism, suggesting a key role of these amino acids in synthesis of various proteins that are required for barley plants growth and development adjustement at the tillering stage. In addition to the classic role of amino acids in the synthesis of proteins, biosynthesis of amino acids such as lysine and isoleucine as well as sulfur/nitrogen containing amino acids like methionine supports plant energy requirements for growth and development [59]. Under energy deficiency, lysine and isoleucine catabolism flux directly into the tricarboxylic acid (TCA) cycle, while methionine is converted into isoleucine [60]. These increased amino acids in leaves of compost-treated plants could lead to enhance physiological processes associated with plant growth such as energy metabolism (TCA cycle), cell division, growth hormones regulation and protein biosynthesis.

### 3.7. Proteins Involved in Protein Metabolism

Changes in protein abundance concerned the proteins related to protein biosynthesis and folding such as plastid-specific ribosomal protein (PSRP) and heat shock protein (HSP), respectively. PSRPs are essential components of protein synthesis machinery that promote efficient protein synthesis and function of chloroplast ribosomes, necessary for cellular growth. Moreover, PSRP have involved in photosynthesis, leaf development and stress resistance, indicating their diverse roles in plant growth and development [61]. Heat shock proteins (HSPs) functioning as molecular chaperones which responsible for protein synthesis, folding, translocation and the degradation of misfolded proteins [62]. HS showed similar results in the activation of proteins related to protein metabolism such as HSPs in Arabidopsis [8] and maize [21]. In this study, compost regulates ribosomal proteins which could lead to stimulate proteins synthesis that are crucial in the current metabolic state, suggesting their key role in many physiological processes such as cell division and proliferation and barley plant development.

### 3.8. Proteins Involved in Biosynthesis of Secondary Metabolites

Compost application induced changes in the abundance of proteins involved in the biosynthesis of secondary metabolites such as Mg-protoporphyrin IX monomethyl ester (oxidative) cyclase (MgPEC) and L-galactose dehydrogenase (LGALDH). These results are consistent with those reported previously. Indeed, the application of PH and HS and other classes of biostimulants induced the expression of genes and the abundance of proteins related to the biosynthesis of secondary metabolites [8,17,21]. MgPEC catalyze the early step of tetrapyrroles biosynthesis such as porphyrins, siroheme, heme, phytochromobilin, chlorophyll a and chlorophyll b. In plants, these tetrapyrroles provide a large number of critical functions such as detoxification of ROS, assimilation of nitrate and sulfate, respiration, photosynthesis and signaling mechanisms that regulate gene expression [63]. In higher plants, L-galactose dehydrogenase catalyses a step in the ascorbate (AsA) biosynthesis. AsA acts as a cofactor for several enzymes involved in multiple cell processes, including hormone biosynthesis (IAA, GA, ABA and ethylene), photosynthesis and respiration, cell division and differentiation, indicating its potential role as a key regulator in plant growth and development and adaptation to stress [64].

### 3.9. Proteins Involved in Hormone Metabolism

In this research, compost application induced changes in the abundance of proteins involved in the biosynthesis and metabolism of phythormones especially jasmonic acid (JA) and abscisic acid (ABA). Allene oxide synthase (AOS) is one of the key enzymes that catalyse the first step of JA biosynthesis [65], weas increased in abundance under compost treatment. Similarly, the abundance of Mg-chelatase, a chloroplast/plastid protein, binds ABA and functions in ABA signaling [66] was increased. These results are consistent with those of previous findings [17,18] which reported that some classes of biostimulants, especially HS and PH induce biosynthetic genes of phythormones including indole-3-acetic acid (IAA), abscisic acid (ABA), salicylic acid (SL), jasmonic acid (JA), gibberellin (GA), cytokinin (CK), brassinosteroid (BR) and ethylene (ET). These results further proved that JA and ABA may play a vital role for regulating barley plant adaptations to biotic and abiotic stresses as well as a number of plant developmental processes [20]. The hormone signaling pathway elicited by compost likely triggers a cascade of phosphorylation events mediated by a variety of protein kinases, which ultimately leads to the transcription of defense-related genes (particularly *PR1*, *JIP-23*, *GLP*, *LEA3*, *HSP*, *ANN7*, and *GST*). Some of these genes are mainly involved in abiotic stress, like *LEA3* and *HSP* but most of them are important in plant defense against pathogens or herbivores.

### 3.10. Proteins Involved in Transport

In this study, three downregulated protein related to solute transport were identified as NRT1/PTR FAMILY, lysine/histidine transporter and tonoplast intrinsic protein. These proteins maintain ion homeostasis, osmotic adjustment, signal transduction and detoxification under stress conditions by facilitating the movement of various molecules and ions through the plasma membrane, improving plant growth performance under abiotic stress conditions [67]. Under optimal conditions solute transport proteins were not significantly represented in barley leaf tissues.

### 3.11. Proteins Related to Epigenetic Regulation Mechanisms

In this study, leaves of barley plants treated with compost showed upregulation of methyltransf_11 domain-containing protein, a methyltransferase type 11 related to the establishment of DNA methylation which play a role in genome management and in regulation of gene expression during plant development as well as for environmental stress responses. These results are consistent with those reported previously in maize [68], tomato [69] and *Salvia miltiorrhiza* [70] plants treated with microbial-based biostimulants. It is possible that the upregulation of methyltransf_11 protein involved in epigenetic regulation by compost treatment induced changes in DNA methylation levels, affecting the abundance of various proteins and the latter could span proteins related to different pathways that may be crucial to barley plant growth and development and tolerance to environmental stresses.

### 3.12. Proteins Related to Other Functional Categories

Adenylosuccinate lyase (ADSL) is an essential enzyme involved in purine metabolism [71]. Thus, the upregulation of this enzyme in leaf tissues greatly influences barley growth and development by increased purine metabolism to generate energy.

Oxidoreductase related proteins have 4 proteins (sanguinarine reductase, short-chain dehydrogenase/reductase 2, ferredoxin and cytochrome P450) under compost treatment. Besides, except the sanguinarine reductase, all other oxidoreductases were downregulated. Cytochrome P450 involved in several processes of plant growth and development also protects plants from abiotic and biotic stresses by the biosynthesis and regulation of several defense compounds [72]. Ferredoxin plays an important role in the photosynthetic rate by regulating electron transfer and chlorophyll content [73]. Moreover, previous studies reported that this enzyme regulates the activities of enzymes associated with periplasmic carbon metabolism [74]. Short-chain dehydrogenases/reductases (SDRs) are involved in either primary or secondary metabolisms including fatty acid and chlorophyll biosynthesis, terpenoids, steroids, phenolics and alkaloids synthesis and metabolism as well as in redox sensor mechanisms [75]. In general, we believe that when the barley plant is under compost treatment, it will synthesize fewer proteins related to REDOX enzymes, reducing the enzyme activity.

## 4. Materials and Methods

### 4.1. Experimental Design and Operations of the Experiment

The experiment was carried out from December 2020 to June 2021 in a field of the production station of ASOC (Association for Saving Oasis of Chenini, Gabes, Tunisia). Physico-chemical analyses of the experimental soil are presented in Table 4.

Experimental treatments were organised in random blocks design (each experimental plot size was 3 m length × 2 m width) with 3 repetitions per treatment as the following: (T1) unamended soil (control) and (T2) soil amended with 30 t ha^−1^ of date palm waste compost. Based in our previously finding, date palm compost at moderate dose (30 t ha^−1^) showed highly beneficial for barley plant growth and yield [9]. Physical, chemical and biological properties of the date palm leaf compost used in this study are presented in Table 5. The plots were spaced 1 m within and between rows. Plots were irrigated once immediately after sowing to ensure uniform emergence and regularly with conventional intervals of 15 days. The local barley (*Hordeum vulgare* L.) cultivar Sahli, which is the most commonly grown cultivar in Tunisia organic farming, was used in this study and seeds were sown at seed rate of 120 kg ha^−1^. Some growth and physiological related parameters were assessed at the tillering stage. For proteomic and molecular analyses, leaves from the same position from thirty different plants were collected randomly for each treatment, mixed separately, snap frozen in liquid nitrogen and stored at −80 °C. For the further analysis, three biological replicates were used.

### 4.2. Agronomic and Physiological Parameters

Ten plants were sampled from each plot and used to measure shoot length (SL) and shoot dry weight (SDW) at the tillering stage. After measurement of SL, plants were separated into root and shoot and then oven-dried at 65 °C for 72 h to determine the biomass. The value of half a gram of fresh leaves was homogenized in a mortar with 5 mL of cold acetone (80% *v*/*v*) to determine the content of photosynthetic pigments. After centrifuging at 10,000 rpm for 15 min, the supernatant of each sample was measured spectrophotometrically at 663, 645 and 480 nm and pigment contents were calculated using the following formulas [76]:

Chlorophyll a (Chla) = 12.70(A_663_) − 2.69(A_645_) (1)

Chlorophyll b (Chlb) = 22.90(A_645_) − 4.68(A_663_) (2)

Total chlorophyll (Chlt) = 20.20(A_645_) + 8.02(A_663_) (3)

Carotenoids (Cart) = A_480_ + 11.40(A_663_) − 63.80(A_645_) (4)

For gas exchange parameters such as net photosynthetic rate (Pn), stomatal conductance (gs), transpiration rate (E) and internal concentration of CO_2_ (Ci) were registered using a portable photosynthesis measurement system (LCpro+, Inc., Hoddesdon, UK). The measurements were taken on 5 completely expanded flag leaves per treatment chosen randomly from 9:00 h to 11:00 h with a photosynthetic photon flux density of 980 µmol m^−2^ s^−1^ during the monitoring.

### 4.3. Total Protein Extraction and Digestion

Proteins were extracted using phenol protocol [77]. Briefly, frozen powder (0.1 g) was vortexed in 10 mL of 50 mM Tris HCl (pH 7.5) containing 0.7 M sucrose, 50 mM EDTA, 0.1 M KCl, 10 mM thiourea, 2 mM phenylmethylsulfonyl fluoride (PMSF) and 2% β-mercaptoethanol. Then, an equal volume of water-saturated phenol was added and the mixture was incubated on a shaker for 30 min at 4 °C. The aqueous and organic phases were separated by centrifuging for 30 min at 12,000× *g* at 4 °C. The phenolic phase was recovered and re-extracted with an equal volume of extraction buffer. Proteins were precipitated by adding 5 volumes of cold methanol containing 0.1 M ammonium acetate and incubated overnight at −20 °C. After centrifuging for 30 min at 10,000× *g* at 4 °C, the precipitated proteins were washed with methanol and then with cold acetone at −20 °C. Proteins were then solubilized in the rehydration buffer (6 M urea, 2 M thiourea, 10 mM DDT, 30 mM Tris HCl pH 8.8 and 0.1% RapiGest (*w*/*v*)). Proteins were quantified using the PlusOne 2D Quant Kit (GE Healthcare, Piscataway, NJ, USA). For digestion, the protein solutions were first incubated at room temperature for 30 min and then alkylated with 50 mM iodoacetamide for 45 min at room temperature in darkness. The protein samples were then diluted 10 times by adding 50 mM ammonium bicarbonate buffer to reduce the urea concentration to 1 M. Finally, trypsin was added at 1:50 trypsin-to-protein mass ratio for the first digestion overnight at 37 °C. After trypsin digestion, the peptide fraction was desalted by Strata X C18 SPE column (Phenomenex) and vacuum dried. Peptides were solubilized in 200 μL of a solution containing 2% acetonitrile and 0.1% formic acid.

### 4.4. Protein LC-MS/MS Analyses

LC-MS/MS was performed using an Eksigent nanoLC Ultra 2D nanoHPLC (SCIEX) coupled to a Q Exactive PLUS mass spectrometer (Thermo, Waltham, MA, USA) at PAPPSO proteome analysis platform (http://pappso.inra.fr, accessed on 26 November 2021). For each sample, about 400 ng of protein digest were loaded onto a Biosphere C18 precolumn (0.1 × 20 mm, 100 Å, 5 μm; Nanoseparation) at 7.5 μL min^−1^ and desalted with 0.1% formic acid and 2% acetonitrile. After 5 min, the pre-column was connected to a Biosphere C18 nanocolumn (0.075 × 300 mm, 100 Å, 3 μm; Nanoseparation). Electrospray ionization was performed at 1.6 kV with an uncoated capillary probe (10 μm tip inner diameter; New Objective, Woburn, MA, USA). Buffers were 0.1% formic acid in water (A) and 0.1% formic acid and 100% acetonitrile (B). Peptides were separated using a linear gradient from 5 to 35% buffer B for 110 min at 300 nL min^−1^. One run took 150 min; including the regeneration step at 95% buffer B and the equilibration step at 100% buffer A [78]. Data-dependent MS analysis was performed with full scans at a 70,000 resolution and MS/MS scans at a 17,500 resolution. The isolation window was repeated for the eight most intense ions detected in full scan and dynamic exclusion was set to 60 s. Xcalibur raw data were transformed to mzXML open-source format using the msconvert software (Version 3.0.19161-f1b0b59b1) in the ProteoWizard 3.0.3706 package [79].

### 4.5. Protein Identification and Quantification

Protein identification was performed using the protein sequence database of *Hordeum vulgare* downloaded from UniProt database (https://www.uniprot.org/, accessed 1 August 2022), with 58,482 entries. Data filtering and protein inference were performed using X!TandemPipeline (version 2015.04.01.1; http://www.thegpm.org/TANDEM/, accessed 1 August 2022). Trypsin digestion was set with one and five possible miss cleavages in the first and refine pass, respectively. Cysteine carbamidomethylation was set as a fixed modification. Methionine oxidation, protein Nter acetylation with or without excision of methionine, Nter glutamine deamidation, Nter carbamidomethyl cysteine deamidation, and Nter glutamic acid dehydration were set as potential modifications. Only proteins identified with at least two independent, unique peptides per protein with an evalue smaller than 0.01were considered [80]. Quantification by integration of the extracted ion current (XIC) was operated using the MassChroQ software [81]. Only proteins quantified with at least 2 specific peptides that were present in at least 90% of the samples were selected for analysis [81].

### 4.6. Bioinformatics and Data Analysis

The proteins identified were mapped to Universal Protein Resource (UniProt) to assess their function [82]. Functional annotations of differentially abundant proteins (DAPs) were performed using Gene Ontology (GO) (http://www.geneontology.org, accessed 1 August 2022). The proteins were classified by Gene Ontology annotation based on three categories: biological process, cellular component and molecular function. Moreover, the DAPs were assigned to various metabolic pathways using the Kyoto Encyclopedia of Genes and Genomes (KEGG) pathway analysis (http://www.genome.ad.jp/kegg/, accessed 1 August 2022).

String web-based program (version 11.5) (http://www.string-db.org/, accessed 1 August 2022) was used to construct a protein interaction network for the identified DAPs. Protein–protein interaction (PPI) networks were then constructed using String software with Confidence Scores greater than 0.7 [83]. The different line colours represent the types of evidence used in predicting the associations: neighbourhood (green), co-occurrence across genomes (blue), co-expression (black), experimental (purple), and association in curated databases (light blue) or texting (yellow). Disconnected nodes or proteins not connected to the main network were hidden in the network.

### 4.7. Total RNA Extraction, cDNA Synthesis and Quantitative Real-Time PCR (qRT-PCR) Analysis

Total RNA was isolated from frozen leaf samples (0.2 g) using a lithium chloride protocol [84]. The residual genomic DNA was removed by RNase-free DNase I (Thermo Fisher Scientific, Waltham, Massachusetts, United States) for 30 min at 37 °C. RNA quality was determined by Nanodrop spectrophotometer by estimating the ratio of absorbance at 260/280 and 260/230 nm and its integrity was checked by electrophoresis in 1.2% agarose gels. The first strand cDNAs were synthesized using revert Aid First Stand cDNA Synthesis Kit (Biomatik; Wilmington, DE, USA) following the manufacturer’s protocol. Based on PPI data, 14 DAPs related to redox homeostasis, plant defense and phytohormone metabolism were selected and gene-specific primers (Table 3) were designed for qRT-PCR using Primer3 Input (version 0.4.0) software [85]. Barley actin was used as an internal control for data normalization. The PCR reactions were performed using the 7300 Real-Time PCR Detection System (Applied Biosystems, Foster City, CA, USA) and the Maxima SYBR Green/ROX qPCR Master Mix (2X) kit (Biomatik; Wilmington, DE, USA) with the following programme: 95 °C denaturation for 10 min, then 40 cycles of denaturation at 95 °C for 30 s, annealing/elongation at 60 °C for 1 min. To confirm that only one PCR product was amplified and detected, a melting curve analysis of amplification products was performed at the end of each PCR by slow heating from 65 °C to 95 °C at 0.5 °C/s and continuous monitoring of the fluorescence signal. Relative expression was calculated using the 2^−ΔΔCt^ method [86].

### 4.8. Statistical Analysis

All the data are presented as means ± standard deviation (SD) of at least three independent experiments. Statistical significance between different treatments was examined by one-way analysis of variance (ANOVA) and Tukey’s Honestly Significant Difference (HSD) test (*p* < 0.05) using Statistical Package for Social Sciences (SPSS Version 20.0) software (SPSS Inc., Chicago, IL, USA).

## 5. Conclusions

Application of date palm waste compost showed effectiveness as biofertilizer in barley plants by increasing plant biomass. Moreover, compost tested in this study caused significant alterations in the abundance of barley leaf proteins. These identified proteins were involved in various metabolic pathways such as photosynthesis, energy metabolism and secondary metabolism. Furthermore, a number of gene-coding proteins have been identified in barley as potential target of compost, such as those involved in defense against biotic and abiotic stress, detoxification of ROS and phytohormones modulation. These results provide more insights into the molecular mechanisms of application of date palm waste compost on barley leaves. Nevertheless, it would be interesting and useful to integrate genomics, proteomics, transcriptomics, and metabolomics analyses to further characterize the potential mechanisms of compost on barley plant growth and development.

## Figures and Tables

**Table 1:** Effect of compost on shoot length (SL), shoot dry weight (SDW), chlorophyll a (Chla), chlorophyll b (Chlb), total chlorophyll (Chlt) and carotenoids (Cart) content, net photosynthetic rate (Pn), stomatal conductance (gs), transpiration rate (E) and internal concentration of CO_2_ (Ci). All data represent means ± standard deviation (SD) of three replicates. Different letters denote significant differences (Tukey’s HSD, *p* < 0.05).

Parameters	Control	Compost
SL (cm)	39.65 ± 0.49 ^b^	51.20 ± 1.8 3 ^a^
SDW (mg plant^−1^)	329.10 ± 7.07 ^b^	536.06 ± 21.22 ^a^
Chla (mg g^−1^ FW)	10.47 ± 0.41 ^b^	12.67 ± 0.37 ^a^
Chlb (mg g^−1^ FW)	2.84 ± 0.13 ^b^	3.28 ± 0.21 ^a^
Chlt (mg g^−1^ FW)	13.32 ± 0.55 ^b^	15.67 ± 0.15 ^a^
Cart (mg g^−1^ FW)	0.38 ± 0.02 ^b^	0.83 ± 0.08 ^a^
Ci (µmol CO_2_ mol^–1^)	282.00 ± 2.82 ^b^	298.00 ± 4.24 ^a^
E (mmol H_2_O m^–2^ S^–1^)	0.49 ± 0.02 ^b^	0.82 ± 0.01 ^a^
gs (µmol H_2_O m^–2^ S^–1^)	49.50 ± 3.53 ^b^	87.50 ± 4.94 ^a^
Pn (µmol CO_2_ m^–2^ S^–1^)	4.87 ± 0.50 ^b^	9.27 ± 0.62 ^a^

**Table 2:** Differentially abundant proteins (DAPs) in barley leaves under compost treatment.

No	Protein Name	Function	Regulated Type	Fold Change	Protein Accession	KEGG Pathway
1	Pathogenesis-related protein 1	Defense response	Up	1.52	P16273	MAPK signaling pathway
2	Ribosome-binding factor PSRP1	Protein biosynthesis	Up	1.52	F2DV60	Other pathways
3	Nucleoside diphosphate kinase 1	Nucleotide metabolism	Up	1.52	A0A287NWM2	Metabolic pathways
4	Magnesium-protoporphyrin IX monomethyl ester (oxidative) cyclase	Chlorophyll metabolism	Up	1.53	F2D891	Biosynthesis of secondary metabolites
5	Adenylosuccinate lyase	Nucleotide metabolism	Up	1.54	A0A287P260	Biosynthesis of secondary metabolites
6	Phenylacetaldehyde reductase	Catalytic activity	Up	1.59	F2DCH1	Other pathways
7	Delta-1-pyrroline-5-carboxylate synthase	Amino acid metabolism	Up	1.62	A0A224MLP5	Biosynthesis of amino acids
8	Allene oxide synthase	Phytohormone action	Up	1.66	F2CWX3	Biosynthesis of secondary metabolites
9	Photosynthetic NDH subunit of lumenal location 2. chloroplastic	Photosynthesis	Up	1.70	F2CTW7	Energy metabolism
10	Sanguinarine reductase	Oxidoreductase	Up	1.73	M0VI12	Metabolic pathways
11	Ribonuclease	RNA processing	Up	1.73	O04393	Other pathways
12	L-galactose dehydrogenase	Redox homeostasis	Up	1.74	F2CWR4	Biosynthesis of secondary metabolites
13	Mg-chelatase	Phytohormone action	Up	1.75	Q94C01	Biosynthesis of secondary metabolites
14	D-ribulose kinase	ATP binding	Up	1.79	F2CZJ1	Carbon metabolism
15	Bifunctional aspartokinase/homoserine dehydrogenase (AK-HSDH)	Amino acid metabolism	Up	1.79	F2EJ93	Biosynthesis of amino acids
16	Phospho-2-dehydro-3-deoxyheptonate aldolase	Amino acid metabolism	Up	1.81	F2D2N1	Biosynthesis of amino acids
17	Aconitase_C domain-containing protein	Clade-specific metabolism	Up	1.84	F2DE08	Citrate cycle (TCA cycle)
18	3-isopropylmalate dehydratase	Clade-specific metabolism	Up	1.86	F2DK00	Biosynthesis of amino acids
19	Group3 late embryogenesis abundant protein	Stress response	Up	1.87	Q43478	Metabolic pathways
20	Methyltransf_11 domain-containing protein	methyltransferase activity	Up	1.98	F2CR07	Metabolic pathways
21	Annexin D7	Stress response	Up	1.99	A0A287UPG1	Other pathways
22	Purple acid phosphatase	Catalytic activity	Up	2.45	F2E6U9	Metabolic pathways
23	23 kda jasmonate-induced protein	Defense response	Up	2.50	P32024	Other pathways
24	Myrosinase-binding protein 2-like	carbohydrate binding	Up	3.14	F2CV07	Other pathways
25	Heat shock protein	Protein homeostasis	Up	3.26	F2DYT5	Protein Processing
26	Glutathione S-transferase	Redox homeostasis	Up	6.67	M0V2H7	Metabolic pathways
27	Translocase of chloroplast 34. chloroplastic-like	Protein translocation	Down	0.06	M0XFE2	Other pathways
28	Ultraviolet-B receptor UVR8-like	External stimuli response	Down	0.23	F2CZC7	Ubiquitin mediated proteolysis
29	Peroxidase (POX)	Response to oxidative stress	Down	0.27	M0Z9D1	Biosynthesis of secondary metabolites
30	Short-chain dehydrogenase/reductase 2	Oxidoreductase	Down	0.39	M0YJ05	Metabolic pathways
31	Eceriferum-c	transferase	Down	0.41	M0XGE3	Biosynthesis of secondary metabolites
32	Thaumatin-like protein (TLP5)	Defense response	Down	0.43	Q5MBN2	Other pathways
33	Cysteine proteinase	Protein homeostasis	Down	0.48	F2DJL2	Other pathways
34	Ferredoxin-NADP reductase	Oxidoreductase	Down	0.52	F2DFN1	Energy metabolism
35	Nicotianamine synthase 9	Nutrient uptake	Down	0.53	Q9XFB7	Biosynthesis of secondary metabolites
36	Glycosyltransferase	Transferase	Down	0.53	F2CUZ3	Metabolic pathways
37	Chitinase	Polysaccharide catabolic process	Down	0.53	A0A287IRA5	Metabolic pathways
38	Germin-like protein	Stress response	Down	0.54	A0A287ERI6	Other pathways
39	Subtilisin-like protease SBT3	Protein homeostasis	Down	0.54	A0A287M050	Other pathways
40	RHOMBOID-like protein	Protein homeostasis	Down	0.55	F2DXL3	Other pathways
41	Pectinesterase	Cell wall organisation	Down	0.55	F2DNY3	Metabolic pathways
42	Cytochrome P450 89A2	Oxidoreductase	Down	0.55	F2DFD7	Other pathways
43	DUF1338 domain-containing protein	Amino acid metabolism	Down	0.55	F2DXZ9	Other pathways
44	Protein NRT1/PTR FAMILY	Solute transport	Down	0.56	F2DQ88	Other pathways
45	Saccharopine dehydrogenase-like protein	Amino acid metabolism	Down	0.56	K9JGW1	Biosynthesis of amino acids
46	Legumain	Multi-process regulation	Down	0.57	B4ESE3	Metabolic pathways
47	Non-specific lipid transfer protein GPI-anchored 1	Cell wall organisation	Down	0.57	F2CSC9	Other pathways
48	Delta tonoplast intrinsic protein 2	Solute transport	Down	0.57	B8R6A6	Other pathways
49	Putative lysine/histidine transporter	Solute transport	Down	0.57	J7RA72	ABC transporters
50	Abhydrolase_3 domain-containing protein	Hydrolase activity	Down	0.58	A0A287KEW5	Metabolic pathways
51	Quinolinate phosphoribosyltransferase	NAD/NADP biosynthesis	Down	0.58	F2ECA5	Metabolic pathways
52	Dirigent protein	plant secondary metabolism	Down	0.60	F2CQ16	Other pathways
53	Lipase_3 domain-containing protein	Lipid metabolism	Down	0.60	A0A287K7Y5	Other pathways
54	Topless-related protein 1	Phytohormone action	Down	0.61	A0A287DXX3	Other pathways
55	Catalase 2	Redox homeostasis	Down	0.61	F2DBE3	Biosynthesis of secondary metabolites
56	Acyl-activating enzyme 16. chloroplastic isoform X1	Lipid metabolism	Down	0.61	A0A287SYP5	Metabolic pathways
57	Lipoxygenase	Phytohormone action	Down	0.62	F2DFM0	Metabolic pathways
58	Pectate_lyase_3 domain-containing protein	hydrolase	Down	0.62	F2D1A5	Metabolic pathways
59	NAD(P)-binding Rossmann-fold superfamily protein	Protein neddylation	Down	0.62	F2DD18	Biosynthesis of secondary metabolites
60	Haloacid dehalogenase-like hydrolase domain-containing protein Sgpp	Carbohydrate metabolism	Down	0.65	F2DPD5	Other pathways
61	Beta-hexosaminidase	Protein modification	Down	0.66	F2E7M4	Metabolic pathways
62	Beta-glucosidase	Hydrolase	Down	0.66	M0UZF4	Biosynthesis of secondary metabolites

**Table 3:** Sequences of primers used for real time quantitative PCR (qRT-PCR).

Gene	Forward and Reverse Primer 5′-3′	Product Size (bp)
Pathogenesis-related protein (*HvPR*)	F: 5′-CTGCTGAAGGAGGTGGAGAC-3′ R: 5′-CCTCGAAGAGCTTGTTCACC-3′	117
Delta-1-pyrroline-5-carboxylate synthase (*HvP5CS*)	F: 5′-GAGACAAGTCCCGTGTTGGT-3′ R: 5′-CCCACGGAGAACCTTAACAA-3′	134
Group3 late embryogenesis abundant protein (*HvLEA3*)	F: 5′-GGTGGAGACGACCCAAGTTA-3′ R: 5′-GTGACAGCCTCGCTTTTCTC-3′	135
Annexin D7 (*HvANN7*)	F: 5′-CCCTATCACCAAGGACCTCA-3′ R: 5′-GGGGCAAGTGAAACACCTTA-3′	85
Heat shock protein (*HvHSP*)	F: 5′-CAAGATCACCATCACCAACG -3′ R: 5′-TAGTTCTCCAGCGCGTTCTT-3′	138
Glutathione S-transferase (*HvGST*)	F: 5′-AAGATGTTCGTGGGCAAAAC-3′ R: 5′-AGGGCCACCTCTAGCTTCTC-3′	86
Peroxidase (*HvPRX*)	F: 5′-TTCGACAAGAAGCAGCTGAA-3′ R: 5′-GTAGATGTGGTCGCGGAAGT-3′	99
Thaumatin-like protein (*HvTLP5*)	F: 5′-ACTGCCCGCCAACATACTAC-3′ R: 5′-GTGCTGGTCTGGTCATCCTT-3′	85
Chitinase IV precursor (*HvCHI4*)	F: 5′-GGACCAATTCCAGCTACTGC-3′ R: 5′-GACTTGATCCCGTCGAAGAA-3′	121
Germin-like protein 5-1 (HvGLP)	F: 5′-TTCAGCATCCATCCAACAAG-3′ R: 5′-TGATCTGGCTGCTGAGAGTG-3′	121
Protein NRT1/PTR FAMILY (*HvNRT1*)	F: 5′-TTCAGTCTCTCCGGCTTGAT-3′ R: 5′-GGAGCATCGTCTCCGAGTAG-3′	93
Topless-related protein 1 (*HvTRP1*)	F: 5′-CGGCAGTGTTTATGTCATGG-3′ R: 5′-GCTCCATTAGGAGGTGTGGA-3′	108
Catalase 2 (*HvCAT2*)	F: 5′-CATGTGATGGATGGATCTGC-3′ R: 5′-TCGACCACATGATCCACAGT-3′	123
Lipoxygenase (*HvLOX*)	F: 5′-ACGACGTCCAAGTCGTAACC-3′ R: 5′-CACGTCCTCCAGGAACATCT-3′	116
Actin (*HvActin)*	F: 5′-CGACAATGGAACCGGAATG-3′ R: 5′-CCCTTGGCGCATCATCTC-3′	56

**Table 4:** Physico-chemical properties of the experimental soil.

Parameters	Value
Clay (%)	5.50
Silt (%)	8.30
Sand (%)	84.40
Soil texture	Sandy
pH	7.50
EC (dS m^–1^)	4.02
Organic matter (%)	0.905
Total organic carbon (%)	0.525
Total N (g kg^–1^ soil)	0.28
Available P (mg kg^–1^ soil)	4.92
Exchange K (mg kg^–1^ soil)	292
Total coliforms (MPN g DW^–1^ soil)	24 × 10^2^
Faecal coliforms (MPN g DW^–1^ soil)	<0.3
Escherichia coli (MPN g DW^–1^ soil)	<0.3
Faecal Streptococci (MPN g DW^–1^ soil)	39 × 10^2^

**Table 5:** Chemical compounds of date palm waste compost.

Parameters	Value
Total organic carbon (%)	18.58
Total N (%)	1.21
C/N	15.36
P (%)	0.54
K (%)	0.95
Ca (%)	8.18
Mg (%)	1.05
Na (%)	0.42
Alkalinity (% CaCO_3_)	11.50
Zn (mg kg^–1^ DW compost)	70.10
Fe (g kg^–1^ DW compost)	70
Mn (mg kg^–1^ DW compost)	130
Cu (mg kg^–1^ DW compost)	11.60
Cd (mg kg^–1^ DW compost)	0.20
Pb (mg kg^–1^ DW compost)	4.15
Cr (mg kg^–1^ DW compost)	11.50
Ni (mg kg^–1^ DW compost)	5.88
Total coliforms (MPN g DW^−1^ compost)	143.33 ± 5.77
Faecal coliforms (MPN g DW^−1^ compost)	120 ± 17.32
Escherichia coli (MPN g DW^−1^ compost)	114 ± 23.79
Faecal Streptococci (MPN g DW^−1^ compost)	114.33 ± 23.8
Salmonella spp. (MPN g DW^−1^ compost)	<0.3
Shigella spp. (MPN g DW^−1^ compost)	<0.3

## Data Availability

Data is contained within the article and Supplementary Material.

## Supplementary Materials

The following supporting information can be downloaded at: https://www.mdpi.com/article/10.3390/plants11233287/s1, Figure S1: Morphological effects of date palm waste compost on shoots and roots of barley plants; Figure S2: Correlation coefficient (R2) of the fold changes between qRT-PCR and LC-MS/MS values; Table S1: The total identified proteins.
