# Supplementary material for: Proteomic Analysis of Barley (Hordeum vulgare L.) Leaves in Response to Date Palm Waste Compost Application
Source: Plants (Basel). 2022 Nov 29;11(23):3287. doi: 10.3390/plants11233287 (PMC9737688; doi:10.3390/plants11233287)
Supplement: Supplementary file 1 [file plants-11-03287-s001.zip › plants-1964764-supplementary.pdf]

Supplemental Figure S1. Morphological effects of date palm waste compost on shoots and roots of barley plants.
Supplemental Figure S2. Correlation coefficient (R^2) of the fold changes between qRT-PCR and LC-MS/MS values.

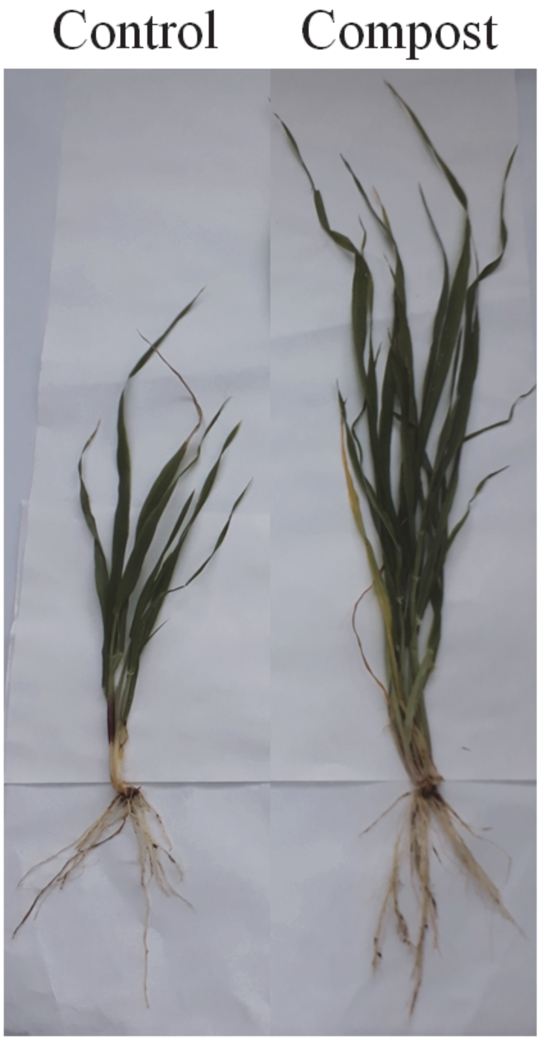

Supplemental Figure S1 Morphological effects of date palm waste compost on shoots and roots of barley plants.

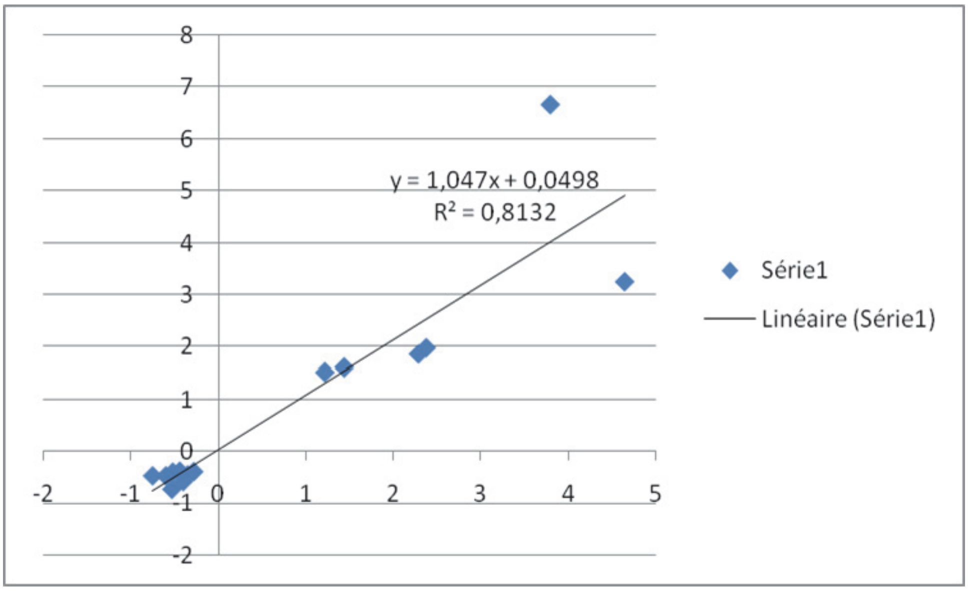

Supplemental Figure S2 Correlation coefficient (R^2) of the fold changes between qRT-PCR and LC-MS/MS values
